# The Diet–Multiple Sclerosis Connection: Oxidative Stress and Emerging Mechanisms

**DOI:** 10.1007/s12035-026-05748-5

**Published:** 2026-02-18

**Authors:** Candida Bucciero, Alessandra Croce, Giuliano Castellano, Francesco Beguinot, Pietro Formisano, Giuseppe Portella, Luca Ulianich, Francesca Fiory, Anna Maria Malfitano

**Affiliations:** 1 Department of Translational Medical Sciences, University of Naples Federico II, 80131 Naples, Italy; 2 Institute of Endotypes in Oncology, Metabolism and Immunology “G. Salvatore”—National Research Council (IEOMI-CNR), 80131 Naples, Italy

**Keywords:** Multiple sclerosis, Oxidative stress, Diets, Epigenome, G-quadruplexes

## Abstract

Multiple sclerosis (MS) is an autoimmune neuroinflammatory disease resulting in myelin degeneration and progressive disability. Oxidative stress plays a crucial role in MS pathogenesis and progression. Nuclear factor erythroid 2-related factor 2 (Nrf2) is a master regulator of the antioxidant mechanisms and its upregulation is associated with beneficial effects in MS. Among the environmental factors influencing MS onset and progression, diet represents a promising non-pharmacological strategy to modulate Nrf2, potentially improving MS outcomes. Indeed, several natural compounds present in Mediterranean, ketogenic and Paleolithic diets can enhance Nrf2 activity, and exert beneficial effects in preclinical models of MS. In this review, we summarize the key role of oxidative stress in MS and highlight how dietary regimens and Nrf2-modulating natural compounds might have therapeutic potential for MS patients. Additionally, we discuss emerging and still poorly explored mechanisms beyond classical Nrf2 activation, including epigenetic regulation and the stability of DNA/RNA secondary structures known as G-quadruplexes, which are involved in gene expression regulation and may represent novel nutrition-based therapeutic targets. However, while Nrf2 modulation by diet is supported by preclinical and limited clinical evidence, targeting G-quadruplexes as a strategy to counteract oxidative stress in MS remains largely speculative and requires further investigation. Notably, epigenetic mechanisms and G-quadruplexes may represent innovative targets of Nrf2-boosting dietary natural compounds for the development of supplemental therapeutic strategies for MS.

## Introduction

Multiple sclerosis (MS) is an immune-mediated, multifactorial chronic demyelinating disease of the central nervous system (CNS), characterized by neurodegeneration, inflammation and axonal loss [1]. The resulting muscle spasticity and weakness lead to motor impairments and a compromised quality of life in MS patients [2]. The pathogenic mechanism involved in MS is characterized by sustained infiltration of autoreactive T cells across the blood–brain barrier into the CNS [3, 4], driving myelin sheath and axon destruction [2].

Oxidative stress (OS) results from excessive formation of reactive oxygen species (ROS), mitochondrial dysfunction and a poor or compromised antioxidant defense system [1]. OS plays a key role in MS, sustaining both the inflammatory and neurodegenerative process of the disease [5]. In the acute phase, OS contributes to the initiation of inflammation, whereas in the chronic phase it sustains neurodegeneration [6].

Intriguingly, plasma isolated from MS patients shows increased levels of OS markers together with altered antioxidant defenses [7]. Autoreactive T lymphocytes induce microglia to release large amounts of ROS, cytokines, oxidative products, and free radicals [8]. Mitochondrial damage occurs at early stages of MS and plays a key role in inflammation and disease progression [9] by increasing the production of toxic ROS, triggering apoptosis, demyelination and neurodegeneration [10]. OS further impairs ATP transport along axons and compromises cellular homeostasis, particularly in neurons and oligodendrocytes, which are notably vulnerable due to weaker antioxidant defenses.

Nuclear factor erythroid 2-related factor 2 (Nrf2), a transcription factor regulating antioxidant gene expression, has emerged as a key modulator of OS in MS. Preclinical studies suggest that Nrf2 activation reduces inflammatory damage and promotes neuronal survival [11, 12]. However, heterogeneity among experimental models and the scarcity of human studies currently limit clinical translation. Interestingly, several dietary regimens, such as Mediterranean, ketogenic, and Paleolithic diets, have been proposed to modulate OS via Nrf2 activation. Despite promising preclinical evidence, clinical outcomes remain inconsistent, as differences in disease stage, patient genetics, and adherence strongly influence efficacy.

This review summarizes the role of OS and Nrf2 in MS and provides a critical synthesis of recent literature on the relevance of diet in MS and the ability of dietary natural compounds in Nrf2 modulation, highlighting updated clinical insights, mechanistic gaps, and translational challenges. Finally, we discuss emerging mechanisms potentially involved in Nrf2 regulation, including epigenetic regulation and the stability of DNA/RNA G-quadruplex structures, which may represent novel nutritional targets to improve the efficacy of current MS therapies.

### Relevance of Nrf2 in MS

Nrf2 orchestrates the cellular antioxidant response through the regulation of genes encoding detoxifying enzymes such as heme oxygenase (HO)−1, NAD(P)H:quinone oxidoreductase 1 (NQO-1), and glutathione S-transferases. Upon exposure to ROS, Nrf2 dissociates from its negative regulator Kelch-like ECH-associated protein 1 (Keap1) and translocates to the nucleus, where it binds to the antioxidant response element (ARE) in the promoter region of these and other genes involved in antioxidant defense and detoxification [13].

Interestingly, several chronic conditions such as respiratory, cardiovascular, kidney, liver and neurodegenerative diseases exhibit an imbalanced Nrf2 pathway [14]. For instance, in Parkinson’s disease (PD), Nrf2 downregulation induces α-synuclein aggregation [15], while Nrf2 activation protects dopaminergic neurons from OS-induced neurotoxicity [16]. In Alzheimer’s disease (AD), the decrease of Nrf2 levels impairs autophagy and leads to the accumulation of amyloid-β and tau [17]. The central role of Nrf2 in these pathologies has prompted investigations aimed at enhancing Nrf2 activity to improve cellular oxidative defense.

In MS, impaired Nrf2 activation is critical for preventing mitochondrial failure, OS, neuroinflammation, and neurodegeneration [18]. Dysfunctions in the Nrf2 pathway disrupt redox homeostasis, leading to increased ROS production [19] and the activation of other redox-sensitive transcription factors, such as the pro-inflammatory nuclear factor kappa-light chain-enhancer of activated B cells (NF-kB) and activator protein 1 (AP-1). Consequently, NF-kB induces the expression of genes involved in MS pathogenesis, such as inducible nitric oxide synthase (iNOS), interleukin 1α/β (IL-1α/β), tumor necrosis factor α (TNF-α), and several growth factors [20]. Additionally, the downstream effectors of Nrf2, such as HO-1, play anti-inflammatory roles in experimental autoimmune encephalomyelitis (EAE) and in MS [21]. In peripheral blood mononuclear cells from MS patients, decreased HO-1 expression correlates with disease exacerbation [22]. However, in autopsy specimens of brain samples and spinal cord from MS patients, Nrf2, HO-1 and NQO-1 were upregulated both within and around active lesions [23], suggesting a role in the endogenous antioxidant defense. Moreover, Nrf2 expression seems to be cell-type-specific [24]: increased levels were observed in macrophages and astrocytes within active lesions [25], and in oligodendrocytes at the lesion’s edges [26]. Notably, damaged oligodendrocytes in EAE lesions exhibit relatively low levels of Nrf2, suggesting their high vulnerability to OS [12, 27]. Consistently, Nrf2-deficient EAE mice display a more rapid disease onset and exacerbated clinical severity, accompanied by enhanced lesions, infiltrating immune cells, higher microglial activation, and visual dysfunction. In contrast, Nrf2 activation correlates with reduced demyelination and neuroinflammation in EAE and other preclinical MS models [28, 29].

Collectively, these studies suggest that strategies to enhance endogenous Nrf2 activation may protect the CNS from oxidative damage. The FDA-approval of dimethyl fumarate (DMF, Tecfidera) [30, 31] as an Nrf2-modulating drug supports the therapeutic relevance of Nrf2 in MS. Upon Nrf2 activation, DMF upregulates the downstream target genes such as NQO1 and HO-1 [32] and induces a shift toward peripheral regulatory immune cells [33], ultimately leading to improved clinical outcomes. Overall, targeting Nrf2 represents a feasible neuroprotective strategy in MS.

### Influence of Diets on Nrf2 Activation

The etiology of MS involves multiple factors including genetic, epigenetic, and environmental determinants. Among the environmental factors, diet is easily modifiable and is known to affect MS progression and symptoms [34]. Indeed, several dietary regimens have been reported to exert beneficial effects in MS as summarized in Table 1. Interestingly, these diets can activate Nrf2, thereby contributing to the induction of antioxidant and anti-inflammatory defenses.

**Table 1:** Dietary regimens beneficial for MS

	Effects in MS	Mechanism/s	References
KD/Fast mimicking diet	Increase of lean mass; improved body composition; reduced depression and fatigue; axon remyelination	Anti-inflammatory; modification of gut microbiota	[93–97]
Paleolithic diet/Swank diet	Improved walking and cognitive performances, fatigue and quality of life	Improvement of serum fatty acids profile	[44, 98, 101–105]
Anti-inflammatory regimen/MD	Improved fatigue, inflammation and clinical manifestations	Anti-inflammatory	[36, 106–109]

The table reports dietary regimens and their beneficial effects in MS along with the type of mechanism elicited

In particular, the Mediterranean diet (MD) and Okinawan diet, which is very similar to Paleolithic diet, are both effective in boosting Nrf2 signaling [35]. The MD is characterized by moderate consumption of fish and dairy products and low intake of meat, in favor of fruit, vegetables and whole grains [36]. The Okinawan diet, considered a modern adaptation of ancestral human diet, is characterized by a high intake of sweet potatoes, vegetables, and plant-based foods, together with a low consumption of meat [35]. In contrast, the Paleolithic diet favors vegetables, fruits, lean meats, fish, and eggs while excluding grains, dairy products, added sugars, and processed foods [37]. The ketogenic diet (KD) has been shown to reduce OS and inflammation by activating Nrf2 in murine models of spinal cord injury [38]. Studies in mice showed that KD initially modifies hippocampal mitochondrial H_2_O_2_ production, which activates the Nrf2 pathway in brain and liver, leading to the induction of protective proteins and the amelioration of the mitochondrial redox state [39]. Similarly, intermitting fasting regimens suppress inflammatory responses by inducing the sirtuin (SIRT) 3/Nrf2/HO-1 pathway in murine models of intracerebral hemorrhage [40]. In addition, fast-mimicking diets, proposed as intermittent nutritional interventions are capable of reducing inflammation and OS by alternating an initial phase of overall caloric restriction with a subsequent phase of normal dietary intake [41, 42]. However, it should be noted that KD and Paleolithic diet may be associated with potential risks, such as nutrient deficiencies, dyslipidemia, and reduced fiber intake [43–45], highlighting the need for careful monitoring and individualized dietary planning in MS patients. These findings suggest a strong rationale for Nrf2-targeting diets; however, translation to humans remains limited due to differences between rodent models and human pathology, variability in diet adherence, and stage-specific responsiveness in MS patients. Moreover, many available studies are limited in sample size, short-term, and lack robust control groups.

### Nrf2-Targeting Dietary Natural Compounds and Their Benefits in MS

Beyond whole dietary patterns associated with Nrf2 activation, recent research identified specific health-promoting natural dietary compounds able to upregulate Nrf2. These compounds belong to different classes of bioactive molecules, including phenolic antioxidants, isothiocyanates from cruciferous vegetables, terpenoids and alkaloids. Several natural compounds, able to regulate Nrf2, have shown beneficial effect in animal models of EAE or demyelination and are currently considered potential dietary supplements for MS. Among these compounds, resveratrol, a natural polyphenol found in several plants, has been shown to activate Nrf2 signaling and promote the expression of antioxidant enzymes such as SOD and glutathione peroxidase, thereby contributing to free radical scavenging and reduction of OS [46]. In lung macrophages, resveratrol modulates Keap1–Nrf2 interaction by targeting the Ile28 residue in DLG motif of Nrf2, inducing conformational changes that alter KEAP1-DLG binding. The disruption of this interaction decreases Keap1-mediated proteasomal degradation of Nrf2, leading to increased Nrf2 expression and nuclear translocation [47]. A similar mechanism has been observed in the brain of AD mouse model, where resveratrol reduces the cytoplasmic Nrf2 levels, while enhancing its nuclear translation rate [48]. In a PD mouse model, resveratrol increases both Nrf2 and SIRT1 expression [49], in turn, SIRT1 activation may indirectly enhance Nrf2 stability via deacetylation-dependent mechanisms [50]. Additionally, in PC12 cells, resveratrol stimulates Nrf2-dependent transcription by activating the Phosphoinositide 3-kinase (PI3K)/Akt and Extracellular Signal-Regulated Kinase (ERK)1/2 pathways [51, 52].

Curcumin, another polyphenol derived from the rhizome of Curcuma longa, activates Keap1-Nrf2-ARE signaling pathway by modifying cysteine sulfhydryl groups in Keap1 [53–55]. Curcumin has also been shown to indirectly activate the ARE system by stimulating the upstream kinases Protein kinase C (PKC) and p38 Mitogen-Activated Protein Kinase (MAPK), which are required for full activation of HO-1, and by inhibiting protein phosphatase activity [56, 57]. Interestingly, two curcumin analogues have also been reported to increase Nrf2 and HO-1 protein expression and promote Nrf2 nuclear translocation, exerting antioxidant and neuroprotective effects in PC12 cells [58]. Notably, the therapeutic potential of curcumin in MS patients has been investigated in several clinical trials [59].

Similarly, several studies reported that the green tea-derived polyphenol epigallocatechin-3-gallate (EGCG) [60] activates the Nrf2 pathway in neuronal [61, 62] and microglial cells [63]. The underlying molecular mechanisms, including activation of ERK and PI3K, have been investigated in different cell types, such as human mammary epithelial cells [64] and hepatocytes [65].

Other natural compounds able to upregulate Nrf2 in the CNS, include matrine, a quinolizidine alkaloid derived from Radix Sophorae Flave [66]; withametelin, a phytosterol isolated from the leaves of datura innoxa plant [67]; piperine, the main bioactive alkaloid of black pepper, sulforaphane, an isothiocyanate found in cruciferous vegetables [68, 69]; myricetin, a flavonoid originally isolated from the bark of the tree Myrica rubra [70]; and many others [71]. Regarding the molecular mechanisms of Nrf2 activation, matrine exerts neuroprotective effects by upregulating Nrf2 via SIRT1 [72], withametelin enhances Nrf2 expression by decreasing Keap-1 levels in the CNS [67], and myricetin induces Nrf2 nuclear translocation in a murine cuprizone-induced demyelination model [73]. Interestingly, recent studies have highlighted the involvement of epigenetic changes in Nrf2 activation [74]. For example, sulforaphane increases Nrf2 expression and nuclear translocation while reducing DNA methylation of the Nrf2 promoter in a cellular model of AD [75]. However, the epigenetic modulation of Nrf2 by dietary compounds remains poorly investigated in the context of MS. Importantly, the beneficial effects of Nrf2-targeting natural compounds have been clearly demonstrated in animal models of demyelination. For instance, resveratrol is among the most extensively studied natural compounds and has shown promising neuroprotective and anti-inflammatory effects in EAE mice, where its supplementation reduced neuroinflammation and ameliorated clinical symptoms [46]. Curcumin also elicits effects comparable to resveratrol on Nrf2 signaling pathway and in MS-related experimental models [76]. Similarly, the Nrf2 upregulation induced by piperine significantly ameliorates memory performance and myelin repair in a rat model of demyelination [77]. Finally, in the cuprizone-induced demyelination model, myricetin improves motor hyperactivity and behavioral deficits [73], and matrine ameliorates clinical signs in EAE [78].

### Potential Mechanisms Beyond Nrf2 Modulation: Epigenome and G-Quadruplexes

Beyond classical Nrf2 activation pathways, emerging evidence highlights that nutritional compounds may influence oxidative homeostasis through additional modulatory molecular mechanisms, including epigenetic regulation and the folding of DNA/RNA G-quadruplex secondary structures, which play key roles in gene expression. Several dietary supplements with antioxidant properties have been reported to act as epigenetic modulators [79]. Interestingly, Nrf2 expression can be influenced by epigenetic mechanisms, such as DNA methylation [74], histone modifications, and interactions with non-coding RNAs [80]. Recent studies have shown that targeting epigenetic modifications within the Nrf2 pathway represents a promising therapeutic strategy using dietary natural compounds such as curcumin, sulforaphane, and resveratrol [79], paving the way for the identification of additional dietary phytochemicals capable of modulating the human epigenome and enhancing antioxidative defense.

To date, the therapeutic potential of G-quadruplex folding modulation has been explored primarily in cancer, where stabilization of these motifs exerts important gene regulatory effects [81–83]. However, direct investigations of G-quadruplex structures in neurodegenerative diseases, including MS, are still at an early stage and warrant further study [84]. Nevertheless, G-quadruplex structures may theoretically affect the Nrf2 pathway through at least two molecular mechanisms. The first mechanism involves the modulation of autophagy, a process essential for the clearance of damaged cellular components and for remyelination following demyelinating injury in MS [85]. Autophagy has been shown to be regulated by G-quadruplexes in neurons [86] and contributes to redox balance by counteracting ROS accumulation via the sequestration and degradation of Keap1, thereby promoting release and activation of Nrf2 [87]. A second potential mechanism involves the presence of G-quadruplex-forming sequences in the promoter region of the Nrf2 gene [88]. Indeed, the 5' untranslated region UTR (5′UTR) of Nrf2 mRNA can adopt G-quadruplex conformations that interact with elongation factor 1 alpha (EF1a), facilitating Nrf2 protein translation under conditions of OS and activating the cellular antioxidant response [89]. Importantly, several dietary natural compounds, including resveratrol, curcumin, and piperine elicit antioxidant effects and can target G-quadruplex motifs [90–92]. In particular, polyphenols have recently been described as G-quadruplex stabilizers of interest for biomedical applications [93–98].

### Translational Limitations of Nrf2-Targeting Nutritional Interventions and Future Perspectives

Dietary natural compounds that boost Nrf2 show promising effects in preclinical MS models by targeting immune regulation, OS suppression, and supporting myelin regeneration. However, clinical trials testing the efficacy of Nrf2-boosting diets and natural compounds, summarized in Table 2, have reported variable outcomes in terms of fatigue amelioration, modulation of metabolic parameters, inflammatory biomarkers, or Magnetic Resonance Imaging (MRI) activity. Despite robust preclinical evidence, differences in study design, disease stage, nutrient dose, and follow-up duration, along with poor bioavailability and lack of standardized formulations, significantly limit clinical translation. For instance, the pronounced lipophilicity of curcumin and resveratrol reduces water solubility, intestinal absorption and systemic exposure [99], which may partly explain the modest or inconsistent outcomes reported in several clinical trials.

**Table 2:** Clinical trials exploring effects of dietary interventions in MS

Intervention (diet or nutrient)	Study design/Trial/Population and size	Main reported outcome(s)	References
MD	Randomized controlled trial, 1-year follow-up; 34 MS patients on Mediterranean-like diet vs 38 on standard healthy diet	↓ fatigue scores; no significant improvement in cognitive status	[110]
KD	18-month randomized controlled trial in 3 groups: 1) KD vs 2) standard diet vs 3) fasting diet in RRMS; 105 enrolled, 81 completed	Primary: no difference in new T2 MRI lesions among groups. Secondary/exploratory: in group 1 improvement in cognition at 18 months; in group 2 ↓ Neurofilament light chain (NfL) at 9 months; cardiometabolic markers improved in groups 1 and 3 and were partially associated with clinical outcomes	[111]
Curcumin (as dietary supplement) add-on to standard therapy	Phase II randomized, placebo-controlled trial (with subcutaneous Interferon β−1 α) in relapsing MS	At 12 months: lower proportion of patients with combined unique active (CUA) MRI lesions in curcumin group vs placebo; at 24 months no significant difference in new/enlarging T2 lesions, relapses, disability, or neurodegeneration markers	[59]
Resveratrol (500 mg/day)	Double-blind randomized placebo-controlled trial, 8 weeks, 55 MS patients (RRMS)	Significant reduction in inflammatory marker (TNF-α) and oxidative stress marker like malondialdehyde (MDA) vs placebo; no change in fatigue score	[112]

The table reports the type of intervention (diet or nutrient) adopted in the clinical trial, the cohort of patients recruited and the main outcomes

Nanoencapsulation has been proposed as a potential strategy to overcome these challenges by protecting natural compounds from degradation, oxidation, and pH extremes, while enhancing solubility, absorption and enabling controlled release. Nevertheless, current research remains focused on evaluating the safety profile of these delivery systems, particularly with respect to long-term exposure and potential toxicity [99]. Additionally, intrinsic differences between animal models and human MS, including disease heterogeneity, treatment duration, dosing regimens, and inter-individual variability, further contribute to the translational gap [100].

These limitations highlight the need for cautious interpretation of preclinical data and underscore the importance of well-designed clinical studies with standardized formulations, optimized delivery strategies, and comprehensive radiological evaluations to accurately assess the therapeutic potential of Nrf2-targeting nutritional interventions in MS.

Furthermore, additional studies are needed to uncover new molecular mechanisms involved in Nrf2 activation that may be targeted by dietary natural compounds. In particular, the potential link between dietary natural compounds, G-quadruplex stabilization, and Nrf2 activation in MS remains entirely hypothetical. To date, no direct experimental or clinical evidence supports a role for G-quadruplex–mediated antioxidant mechanisms in MS models or patients. Exploring these mechanisms may also help identify natural dietary compounds that specifically target G-quadruplexes, potentially paving the way for innovative nutrition-based therapeutic strategies in MS.

## Conclusions

It is well established that Nrf2 is a master regulator of cellular antioxidant defense. To date, numerous dietary natural compounds have been identified for their ability to activate Nrf2 and for their beneficial effects in supporting myelin integrity and reducing neuroinflammation in preclinical models of MS. In this review, we summarized experimental studies highlighting the relevance of the Nrf2 signaling pathway in MS and discussed the Nrf2-targeting properties of specific dietary regimens, including Mediterranean, ketogenic, and Paleolithic diets. We also focused on specific dietary natural compounds able to activate Nrf2 that are commonly consumed in daily diet and have demonstrated beneficial effects in MS-related experimental models. Interestingly, some of these compounds can modulate the epigenome and potentially influence G-quadruplex folding. Notably, while dietary modulation of Nrf2 is supported by experimental evidence, the involvement of G-quadruplex-mediated mechanisms in MS remains largely theoretical, with current insights derived primarily from other biological contexts. Therefore, mechanistic studies and well-designed clinical trials are required to validate these emerging targets and to optimize diet-based interventions as supplemental therapies for MS. To date, robust randomized controlled trials are still lacking, and major challenges related to target selectivity and poor bioavailability of dietary compounds remain unresolved. In conclusion, from a future perspective, combining conventional pharmacological approaches with targeted dietary strategies may represent a promising approach to reduce OS and improve both the efficacy and safety of current MS therapies.

## Data Availability

No datasets were generated or analysed during the current study.
